# Indications for resection of recurrent lesions in patients with distal cholangiocarcinoma based on prognostic factors: a single-institute retrospective study and brief literature review

**DOI:** 10.1186/s12893-022-01879-3

**Published:** 2022-12-12

**Authors:** Taro Mashiko, Toshihito Ogasawara, Yoshihito Masuoka, Shigenori Ei, Shinichiro Takahashi, Masaki Mori, Kazuo Koyanagi, Seiichiro Yamamoto, Toshio Nakagohri

**Affiliations:** Department of Gastroenterological Surgery, Tokai University School of Medicine, 143 Shimokasuya, Isehara, Kanagawa 259-1193 Japan

**Keywords:** Distal cholangiocarcinoma, Prognostic factors, Surgery for recurrence

## Abstract

**Background:**

To evaluate the effectiveness of surgery for recurrent distal cholangiocarcinoma and determine surgical indications based on prognostic factors for the recurrence of distal cholangiocarcinoma.

**Methods:**

We analysed the outcomes of 101 patients who underwent surgical resection for distal cholangiocarcinoma between 2000 and 2018. The clinicopathological factors and prognosis of primary and recurrent distal cholangiocarcinoma were investigated.

**Results:**

Of the 101 patients with resected distal cholangiocarcinoma, 52 (51.5%) had relapsed. Seven (13.5%) and 45 patients (86.5%) underwent resection of recurrent lesions and palliative therapy, respectively. There were no major complications requiring therapeutic intervention after metastasectomy. The median overall survival in patients with and without surgery for recurrent lesions was 83.0 (0.0–185.6) and 34 months (19.0–49.0), respectively. Therefore, patients who had undergone surgery for recurrent lesions had a significantly better prognosis (p = 0.022). Multivariate analyses of recurrent distal cholangiocarcinoma revealed that recurrence within one year was an independent predictor of poor survival. Resection of recurrent lesions improved prognosis.

**Conclusions:**

Radical resection in recurrent distal cholangiocarcinoma may improve the prognosis in selected patients. Although time to recurrence is considered an important factor, the small number of cases of recurrence and resection of recurrent lesions in this study makes it difficult to conclude which patients are best suited for resection of recurrent lesions. This issue requires clarification in a multicentre prospective study, considering patients’ background, such as the recurrence site and number of metastases.

## Background

Cholangiocarcinoma accounts for 3% of all gastrointestinal malignancies [1], while distal cholangiocarcinoma (DCC) accounts for 30% of all bile duct cancers [2]. The only curative treatment for DCC is surgical resection; however, even when an R0 resection is achieved, the recurrence rate is high, and the disease is associated with a poor prognosis. More than 50% of cases recur within 3 years [3, 4] and the 5-year survival rate is 18–54% [5–7].

Chemotherapy is the first choice of treatment for recurrent biliary tract cancers. On the other hand, some reports have suggested that resection of the recurrent lesions improves the prognosis [8–13]. Currently, there is no evidence concerning the resection of recurrent biliary tract cancers, and the indications for surgery have not yet been established.

The aim of this study was to evaluate the outcomes of resection of recurrent DCC in our department and the indications for surgical resection of recurrent lesions based on the prognostic factors of primary and recurrent DCC.

## Methods

### Patients

We enrolled 115 patients who underwent surgical resection for DCC at Tokai University Hospital between January 2000 and December 2018. The standard treatment for DCC is pancreatoduodenectomy (PD); however, bile duct resection (BDR) was chosen at the discretion of the attending physician for older patients or those for whom there were concerns regarding their ability to tolerate PD, and when the main lesion was in the middle bile duct. Staging of the primary DCC was performed according to the Union for International Cancer Control Tumor Node Metastasis (UICC TNM) classification, 7th edition. Postoperative complications were evaluated by the Clavien–Dindo classification. One surgery-related death and 13 deaths from other diseases were excluded, and 101 patients were finally analysed. Extensive cholangiocarcinoma treated with hepatectomy with concomitant PD was not considered in this study. The present study was approved by the Institutional Ethical Board of Tokai University Hospital.

### Follow-up after surgery for primary distal cholangiocarcinoma

All patients received routine postoperative surveillance. Patients underwent tumour marker measurements every three months. Chest and abdominal computed tomography (CT) were performed every 3 months for the first 3 years, every 6 months for the following 2 years, and annually thereafter. If necessary, magnetic resonance imaging or positron emission tomography CT was added to diagnose recurrence. Recurrence was diagnosed based on the radiological or pathological findings, and patients with only elevated tumour markers were not considered to have recurrence. Since there is currently no evidence for postoperative adjuvant chemotherapy, the decision to introduce adjuvant chemotherapy was made at the discretion of the attending physician.

### Indications for repeat surgery in recurrent distal cholangiocarcinoma

In our department, the following criteria are defined as indications for resection of recurrent lesions: (1) the patient’s general condition is good; (2) the lesion is a single lesion, or two or more lesions are localised and curatively resectable; and (3) the time to recurrence is more than one year. Patients who do not meet these conditions may be treated with systemic chemotherapy or palliative care, depending on their general condition.

### Statistical analysis

All statistical analyses were performed using a standard statistical programme (SPSS software for windows, version 26.0; Chicago, IL, USA). Chi-square tests were used to analyse categorical variables and Mann–Whitney U tests were used to analyse continuous variables. Overall Survival (OS) and recurrence-free survival (RFS) were analysed using the Kaplan–Meier method, and statistical significance was evaluated by the log-rank test. Univariate and multivariate Cox proportional hazard regression analyses were performed on variables to identify prognostic factors for primary DCC. Multivariate analyses were performed on variables with p < 0.05 from the univariate analyses. A p-value < 0.05 was considered statistically significant.

## Results

### Baseline and clinicopathological characteristics of patients with primary distal cholangiocarcinoma

This study included 101 resected cases of DCC; 84 males and 17 females, with a median age of 72 years (16–86 years). The median observation period was 48 months (2–171 months). Baseline characteristics and clinicopathological features of these 101 patients at the time of surgical resection of primary DCC are shown in Table 1.

**Table 1:** Baseline and clinicopathological characteristics of patients with primary DCC

Variables	All patients (n = 101)
Age, years, median (range)	72.0 (16–86)
Sex	
Male	84 (83.2)
Female	17 (16.8)
Albumin g (d/l)	4.1 (2.2–5.1)
CEA (ng/mL), median (range)	3.2 (1.2–14.4)
CA19-9 (U/mL), median (range)	53.5 (1.0–4815.2)
Surgical procedure	
PD	94 (69.3)
BDR	7 (6.9)
Portal vein resection	5 (5.0)
Operation time (min) median (range)	298 (183–592)
Blood loss (ml) median (range)	761 (114–6814)
Blood transfusion	24 (23.8)
Complication (Clavien–Dindo classification)	
1,2	36 (35.6)
3,4	65 (64.4)
Primary tumour size (mm), median (range)	24 (1.0–90.0)
Gross type	
Papillary	22 (21.8)
Nodular	41 (40.6)
Flat	38 (37.6)
Pancreatic invasion	
Negative	50 (48.5)
Positive	51 (51.5)
Duodenal invasion	
Negative	84 (83.2)
Positive	17 (16.8)
Portal vein incision	
Negative	97 (96.0)
Positive	4 (4.0)
Lymphovessel invasion	
Negative	31 (27.7)
Positive	73 (72.3)
Vascular invasion	
Negative	40 (39.6)
Positive	61 (60.4)
Perineural invasion	
Negative	21 (20.8)
Positive	80 (79.2)
pT	
T1	12 (11.9)
T2	29 (28.7)
T3	60 (59.4)
pN	
N0	69 (68.3)
N1	32 (31.7)
Residual tumour status	
R0	75 (74.3)
R1	26 (25.7)
Tumour differentiation	
Well	59 (58.4)
Moderate	28 (27.7)
Poorly	13 (12.9)
Adenosquamous	1 (1.0)
Adjuvant chemotherapy	
Tegafur/Uracil	2 (5.9)
Gemcitabine	5 (14.7)
S-1	14 (41.2)
Gemcitabine + S-1	13 (38.2)

*CEA* carcinoembryonic antigen, *PD* Pancreaticoduodenectomy, *BDR* Bile duct resection, *DCC* distal cholangiocarcinoma

PD and extrahepatic BDR were performed in 94 (93.1%) and seven cases (6.9%), respectively. Five patients (5.0%) underwent portal vein resection and reconstruction. The median operative time was 298 min (183–592), and the median blood loss was 761 mL (114–6814 mL). Blood transfusion was performed in 24 patients (23.8%), and Clavien–Dindo classification of 3a or higher complications were observed in 65 patients (64.4%), which were attributed to pancreatic fistula grade B. Recent reports have reported an increased incidence of pancreatic fistula above grade B after PD for DCC ranging from 26.3% to 56.0% [14–16]. The high incidence of complications has been attributed to this; fortunately, there were no deaths due to haemorrhage or sepsis. One factor that contributed to this may be the high number of young, relatively inexperienced surgeons who were in training and performed PD in our department.

The median length of stay was 30 days (9–100). There were no in-hospital deaths, but one case of early recurrence resulted in death within 90 days. This patient demonstrated an early rise in tumour markers and abdominal contrast-enhanced CT showed multiple liver metastases that had not been detected preoperatively. It was a case of very early recurrence. The median tumour diameter was 24 mm (1–90). Tumour tissues were well differentiated adenocarcinoma, moderately differentiated, poorly differentiated, and adenosquamous carcinoma in 59 (58.4%), 28 (27.7%), 3 (3.0%), and 1 case (1.0%), respectively. Lymphovessel, venous, and perineural invasions were observed in 73 patients (72.3%), 61 (60.4%), and 80 (79.2%) patients, respectively. Pancreatic and portal vein invasions were observed in 51(50.5%) and four (4.0%) cases, respectively. Lymph node metastasis was observed in 32 patients (31.7%), R0 resection was performed in 75 patients (74.3%), and among the R1 resections, seven patients (26.9%) had carcinoma in situ (CIS). In these cases, additional resection of the bile duct stump on the liver side was performed due to findings of invasive carcinoma or CIS in the bile duct stump at the time of initial resection or additional resection. However, these are cases where the marginal margins for additional resection in the extrahepatic bile duct were still CIS-positive. All patients were ≥ 75 years old, and additional hepatectomy was determined to be difficult in terms of surgical tolerance. No additional hepatectomy was performed, as several reports have shown that positive CIS at the bile duct stump in cholangiocarcinoma has a relatively favourable long-term prognosis [17, 18]. Postoperative adjuvant chemotherapy was administered in 34 patients (33.7%). The regimens were as follows: Tegafur/Uracil in two patients (5.9%), gemcitabine in five patients (14.7%), S-1 in 14 patients (41.2%), and gemcitabine combined with S-1 in 13 patients (68.2%).

### Recurrence and survival

**Fig. 1:**
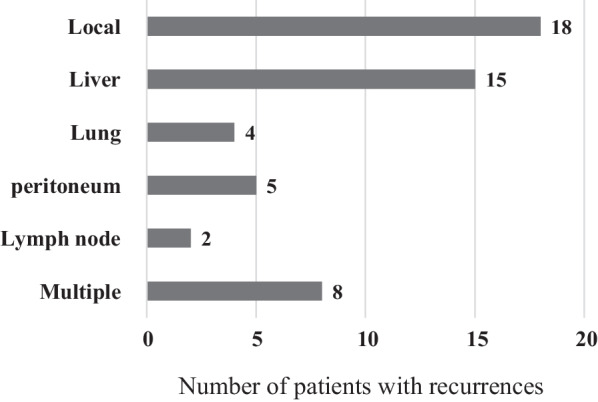
Recurrence site after surgery for primary DCC. *DCC* distal cholangiocarcinoma

**Fig. 2:**
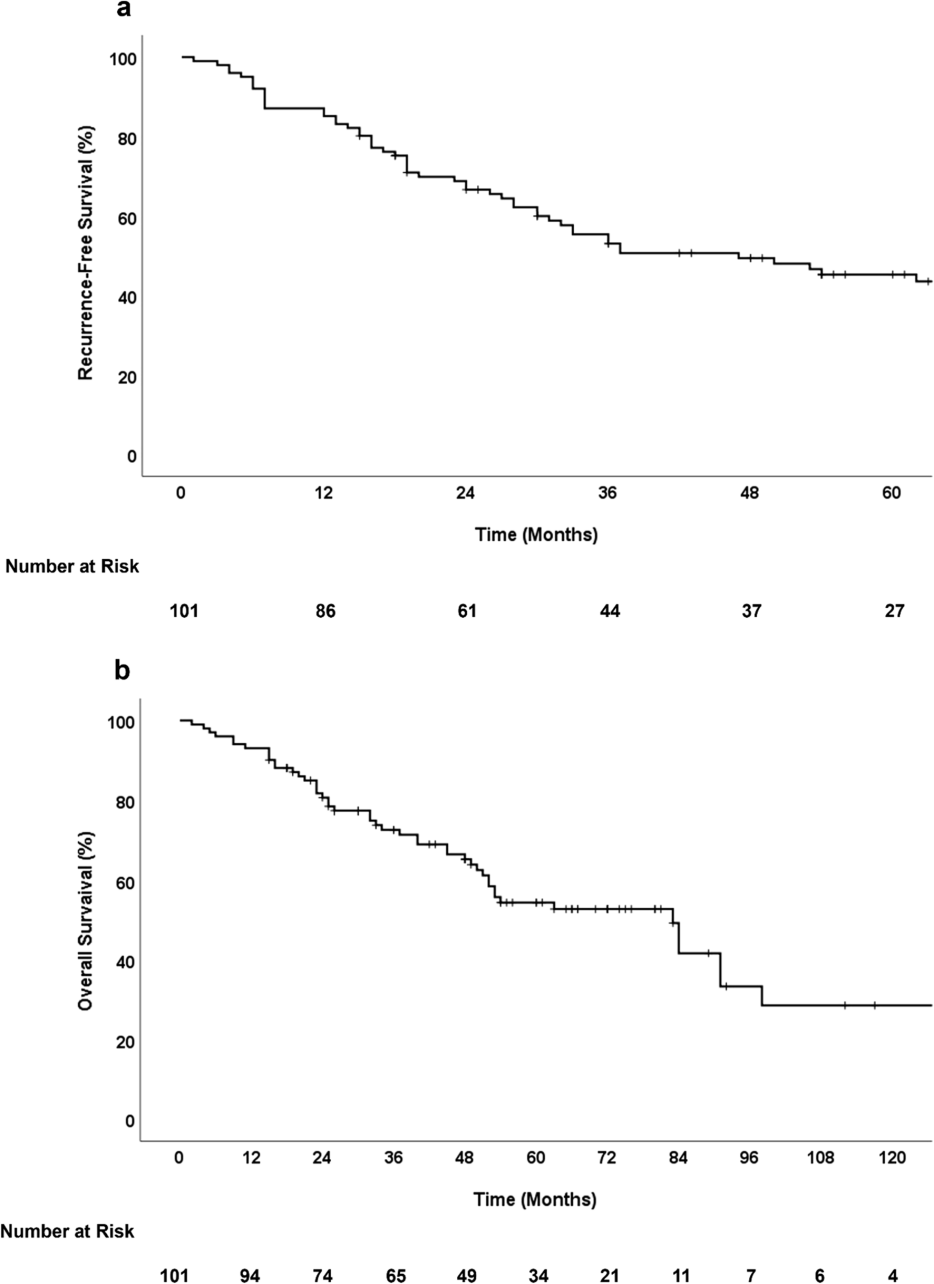
Kaplan–Meier survival curve of patients with primary DCC who underwent resection. **a** Disease-Free survival, **b** Overall Survival. *DCC* distal cholangiocarcinoma

During the observation period, recurrence occurred in 52 of the 101 patients (51.5%). The first sites of recurrence were local, liver metastasis, peritoneal dissemination or abdominal wall recurrence, lung, para-aortic lymph node, and combined recurrence in 18 (34.6%), 15 (28.8%), 5 (9.6%), 4 (7.7%), 2 (3.8%), and 8 (15.4%) patients, respectively (Fig. 1). The median RFS was 47.0 months (21.9–72.1), and the 1, 3, and 5-year RFS rates were 85.1%, 53.1%, and 45.3%, respectively (Fig. 2a). The median OS was 83.0 months (56.3–109.7), with 1, 3, and 5-year survival rates of 93.1%, 72.7%, and 54.4%, respectively (Fig. 2b). There was no significant difference in the OS by recurrence sites (p = 0.237).

### Comparison of clinicopathological factors and recurrence sites in R0 and R1 cases

A comparison of clinicopathological factors between R0 and R1 cases indicated that the tumour diameter was significantly larger in R1 cases (p = 0.001). In addition, the gross type was flat or nodular (p = 0.034), positive pancreatic invasion (p = 0.030), positive portal vein invasion (p = 0.004), positive perineural invasion (p = 0.009), pathological T3 (p < 0.001) were significantly more common in R1 cases. In summary, R1 cases tended to have higher oncological grades. A comparison of recurrence sites revealed that local recurrence was significantly more common in R1 cases (p < 0.001); no significant differences were found for other sites (Table 2). The median RFS did not reach the median for R0 cases, whereas it was 16 months (9.9–22.1) for R1 cases, with R0 cases having a significantly better prognosis (p < 0.001). Similarly, the median OS of R0 patients was 91 months (81.6–100.4) compared with 33 months (8.3–57.7) for R1 patients, with R0 patients having a significantly better prognosis (p < 0.001).

**Table 2:** Comparison of clinicopathological factors and recurrence sites in R0 and R1 cases

Variables	R0 (n = 75)	R1 (n = 26)	p-value
CEA (ng/mL), median (range)	3.2 (1.2–14.4)	4.0 (1.6–11.9)	0.099
CA19-9 (U/mL), median (range)	48.4 (1.0–4815.2)	58.2 (10.8–1253.5)	0.255
Gross type (Papillary)	20 (26.7)	2 (7.7)	0.034
Primary tumour size (mm), median (range)	20 (1–70)	31 (7–90)	0.001
Pancreatic invasion (positive)	34 (45.3)	18 (69.2)	0.030
Duodenal invasion (positive)	11 (14.7)	6 (23.1)	0.242
Portal vein invasion (positive)	0	4 (15.4)	0.004
Lymphovessel invasion (positive)	54 (72.0)	19 (73.1)	0.566
Vascular invasion (positive)	43 (57.3)	18 (69.2)	0.202
Perineural invasion (positive)	55 (73.3)	25 (96.2)	0.009
pT3	36 (48.0)	23 (88.5)	< 0.001
pN1	21 (28.0)	11 (44.0)	0.135
Tumour differentiation (Well)	44 (58.7)	15 (57.7)	0.555
Recurrence site			
Local	7 (9.3)	11 (42.3)	< 0.001
Liver	12 (16.0)	3 (11.5)	0.423
Lung	3 (4.0)	1 (3.8)	0.728
Peritoneum	3 (4.0)	2 (7.7)	0.383
Lymph node	2 (2.7)	0	0.550
Multiple	5 (6.7)	3 (11.5)	0.344

*CEA* carcinoembryonic antigen

### Outcomes of treatment in recurrent distal cholangiocarcinoma

In the 52 patients who relapsed, 7 (13.5%), 28 (53.8%), and 17 (32.7%) underwent resection of recurrent lesions, chemotherapy, and transferred to palliative care, respectively. First-line chemotherapy was administered to 15, 6, 6, and one patient with gemcitabine plus cisplatin, gemcitabine, S-1, and gemcitabine plus S-1, respectively. Resection of recurrent lesions included liver metastasis, abdominal wall recurrences, and PD for local recurrence after extrahepatic BDR in five, one, and one case, respectively. The median age was 75 years (63–79), and all patients were male. All patients had pathological T2 or less, six of the seven cases were negative for lymph node metastasis and R0 resection was performed. At the discretion of the attending physician, resection was performed in only one case of recurrence within one year. Only two patients had resection of second recurrent lesions.

**Fig. 3:**
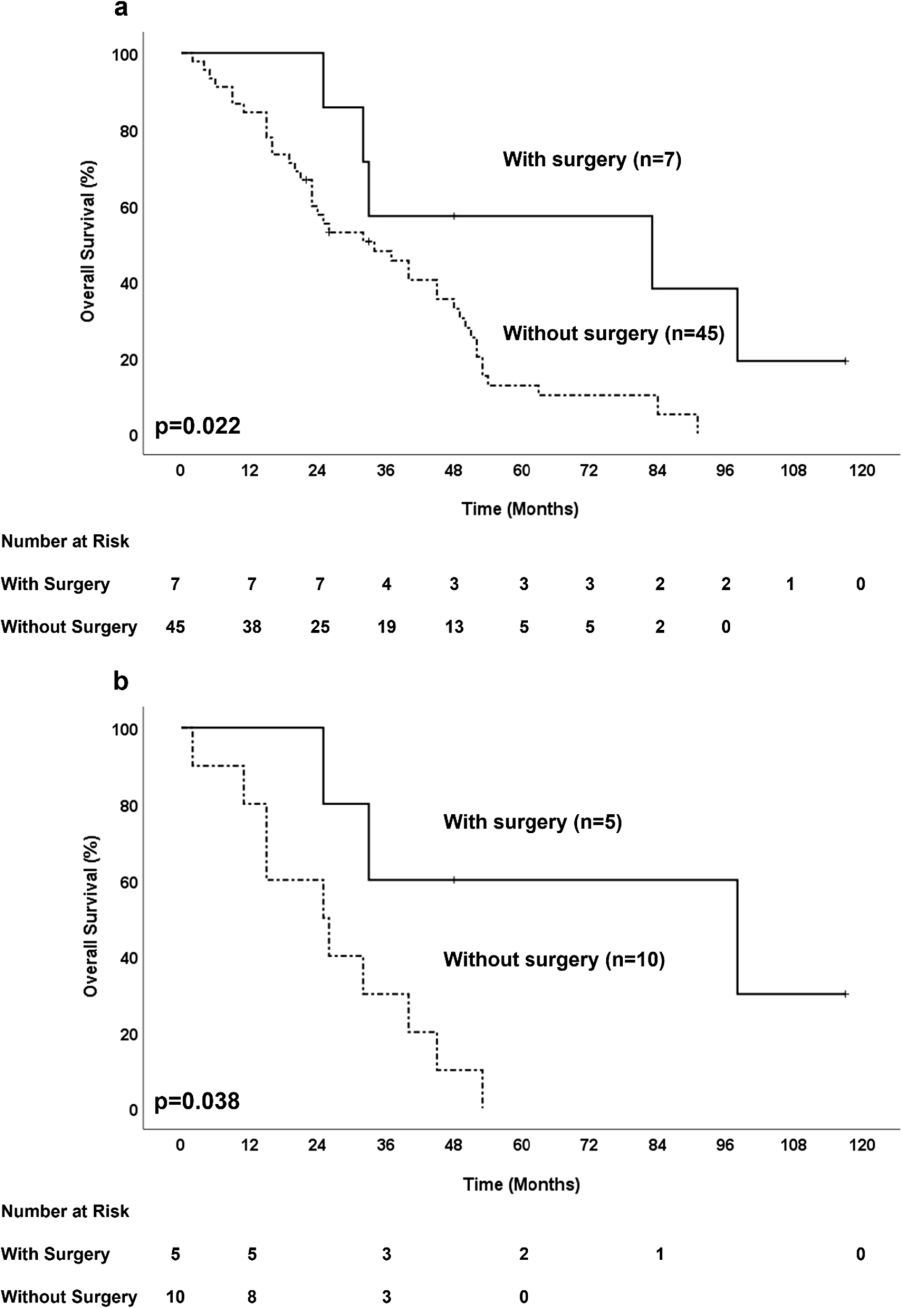
Kaplan–Meier survival curves of patients with recurrent DCC according to the resection of recurrent lesion. *DCC* distal cholangiocarcinoma

There were no cases of recurrent lesion resections requiring perioperative blood transfusion, and there were no complications requiring therapeutic intervention of the Clavien–Dindo classification 3a or higher (Table 3). Median RFS was 19.0 months (16.4–21.6 months), and the median OS was 83.0 months (0.0–185.6 months). The median OS of patients without surgery for recurrent lesions was 34 months (19.0–49.0 months), and the prognosis of patients with surgery was significantly better (p = 0.022) (Fig. 3a). Similarly, the median OS after recurrence was 30 months (19.7–40.3 months) in the resection group, and 10 months (6.0–14.0 months) in the non-resection group, with the resection group having a significantly better prognosis (p = 0.005). A patient who underwent additional PD for local recurrence after extrahepatic BDR died of early local recurrence. In contrast, four of the seven patients achieved a five-year survival. In patients with recurrent liver metastases, the prognosis of patients with resected liver metastases was significantly better than that of patients without resected liver metastases, with an OS of 98 months (0.0–199.4 months) and 26 months (23.1–28.9 months), respectively (p = 0.038) (Fig. 3b).

**Table 3:** Cases of resection for recurrent lesions in distal cholangiocarcinoma

Case	Age/Sex	Primary DCC surgery	Tumour gross type	Primary tumour Size (mm)	pT	pN	R	Tumour differentiation	Adjuvant chemotherapy	RFS (Months)	Recurrence site (tumour number)	Surgical procedure	Second recurrence site	Second recurrence treatment	OS (Months)
1	77 M	PD	Papillary	10	2	0	0	Papillary, Well	–	16	Liver(S5) (1)	Partial liver resection	Liver (multiple)	(BSC)	25 (Death)
2	79 M	BDR	Nodular	3	2	0	0	Poorly	–	30	Local	SSPPD	Local	(BSC)	32 (Death)
3	63 M	PPPD	Papillary	25	1	1	0	Papillary, Well	GEM	53	Abdominal wall (1)	Abdominal wall resection	Lung (1)	Partial lung resection	83 (Death)
4	70 M	SSPPD	Papillary	10	1	0	0	Moderate	–	19	Liver (S5) (1)	Partial liver resection	Unknown	–	98 (Death)
5	75 M	SSPPD	Papillary	5	2	0	0	Papillary, Well	–	28	Liver (S5, S6) (2)	Partial liver resection	–	–	117 (Alive)
6	76 M	SSPPD	Flat	17	3	0	1 (CIS)	Moderate	–	7	Liver (S5, S8) (2)	Partial liver resection	Liver (multiple)	GEM + Cisplatin	33 (Death)
7	71 M	SSPPD	Flat	20	2	0	0	Moderate	–	18	Liver (S6) (1)	Partial liver resection	Lung (1)	Partial lung resection	61 (Alive)

*DCC* distal cholangiocarcinoma, *PD* pancreaticoduodenectomy, *PPPD* pylorus-preserving pancreaticoduodenectomy, *SSPPD* subtotal stomach preserving pancreaticoduodenectomy, *BDR* bile duct resection, *CIS* carcinoma in situ, *GEM* gemcitabine, *RFS* recurrence-free survival, *OS* overall survival, *BSC* best supportive car

### Univariate and multivariate analyses of the prognostic factors in primary DCC

Analysis of prognostic factors in the 101 resected cases of DCC was performed. In univariate analysis, operative time, blood transfusion, tumour size, tumour gross type, portal vein invasion, lymphovessel invasion, venous invasion, perineural invasion, lymph node metastasis, residual tumour status, UICC-T factor, tumour differentiation, and recurrence within one year were significantly associated with prognosis. Multivariate analysis of the prognostic factors showed that positive portal vein invasion (hazard ratio [HR]: 6.44, 2.0–21.1, p = 0.002), positive lymph node metastasis (HR: 2.75, 1.4–5.2, p < 0.001), and recurrence within one year (HR: 30.77, 11.5–82.4, p < 0.001) were independent poor prognostic factors.

### Univariate and multivariate analyses of prognostic factors in recurrent DCC

Analysis of prognostic factors in the 52 recurrent cases of DCC was performed. In univariate analysis, tumour size, tumour gross type, UICC-T factor, and recurrence within one year were significantly associated with prognosis. Multivariate analysis of prognostic factors showed that recurrence within one year (HR: 16.4, 6.5–41.4, p < 0.001) was an independent, poor prognostic factor. Resection of recurrent lesions improved prognosis (HR: 0.29, 0.1–0.9, p = 0.025). In recurrent cases, lymph node metastasis and portal vein invasion were not independent poor prognostic factors (Table 4).

**Table 4:** Prognostic factors of OS in recurrent DCC by univariate and multivariate analyses

Factors	Univariate analysis		Multivariate analysis	
	HR	p-value	HR (95% CI)	p-value
Age (> 73)	0.90	0.736		
Sex (Female)	0.93	0.875		
CEA (> 5.0 ng/ml)	1.00	0.987		
CA19-9 (> 71.5 U/ml)	0.82	0.525		
Operation time (> 306 min)	1.19	0.559		
Blood loss (> 844 ml)	0.68	0.209		
Blood transfusion (Yes)	1.32	0.378		
Clavien–Dindo classification (≥ 3a)	0.99	0.963		
Tumour Size (> 28 mm)	2.21	0.008		
Gross tumour type (Nodular/Flat)	2.59	0.048		
Pancreatic invasion (positive)	1.62	0.116		
Duodenal invasion (positive)	0.98	0.883		
Portal vein invasion (positive)	1.93	0.215		
Lymphovessel invasion (positive)	1.45	0.317		
Venous invasion (positive)	1.93	0.055		
Perineural invasion (positive)	2.32	0.163		
R1 resection	1.77	0.062		
Tumour differentiation (moderate/poor/others)	1.38	0.268		
pT3	2.25	0.019		
Lymph node metastasis (positive)	1.46	0.200		
Adjuvant chemotherapy (No)	0.58	0.077		
Time to recurrence (≤ 12 months)	11.56	< 0.001	16.4 (0.52–41.4)	< 0.001
Resection of recurrent lesions (Yes)	0.30	0.023	0.29 (0.1–0.9)	0.025

*CEA* carcinoembryonic antigen, *HR* hazard ratio, *CI* confidence interval

## Discussion

According to a report of 18,606 cases in the Japanese Biliary Tract Cancer Registry, the 5-year survival rate of all cases was 39.8%, 24.2%, 39.1%, and 61.3% for the gallbladder, perihilar bile duct, distal bile duct, and ampullary cancers, respectively [6]. The 5-year survival rate for intrahepatic cholangiocarcinoma is reported to be 15–40% [19–21]. However, since the prognoses and prognostic factors of intrahepatic cholangiocarcinoma, hilar cholangiocarcinoma, distal cholangiocarcinoma, and gallbladder cancer are slightly different, we thought it would be better to consider them separately, since their biological behaviours are different [8]. Most reports on this topic concern the recurrent resection of all biliary tract cancers or repeat liver resection of intrahepatic cholangiocarcinoma [9–13, 21, 22]. In this study, we focused on DCC as there are few reports on the resection of recurrent lesions in DCC alone.

Various reports indicate that liver metastases are the most common recurrence site of DDC, followed by local recurrence, peritoneal dissemination, lymph node recurrence, and lung recurrence, while bone and other sites of recurrence are relatively uncommon [4, 9, 11, 23, 24]. In our department, local recurrence was more common than liver metastases, but this may be due to the relatively high number of R1 cases (25.7%). However, R1 resection rates were not extremely high, ranging from 3.8% to 22.9% in recent reports [14, 24–26]. Koyama et al. reported that the prognoses for local and distant metastases were comparable (p = 0.975) [4]. The median survival for cases with local recurrence was 34 months, liver metastases 32 months, peritoneal dissemination 15 months, lung metastases 51 months and combined recurrence 9 months, with a relatively better prognosis for lung metastases, but no significant difference in prognosis by the site of recurrence in our study. Conversely, single distant metastases, especially single liver metastases, were reported to have a better prognosis than local or combined recurrences [27].

Systemic chemotherapy is usually the first choice of treatment for recurrent biliary tract cancer. A randomised controlled clinical trial has made gemcitabine combined cisplatin therapy the first-line treatment for unresectable or recurrent biliary tract cancer [28].

According to these reports, patients with unresectable or recurrent biliary tract cancer treated with chemotherapy have a poor prognosis with a median survival of approximately 12 months [28–30]. In recent trials of gemcitabine-based chemotherapy for locally advanced or recurrent biliary tract cancer, a phase II trial of gemcitabine plus nab-paclitaxel therapy showed a median OS of 12.4 months and a median progression-free survival of 7.7 months [31]. In a phase II study of gemcitabine, irinotecan, and panitumumab in advanced biliary tract cancer, the median OS was 12.9 months, and the median progression-free survival was 9.7 months [32]. Bisello et al. reported outcomes of radiotherapy and chemoradiation for unresectable biliary tract cancer, with median OS and PFS of 13.5 and 10 months, respectively [33]. In any case, the prognosis is unsatisfactory. The median OS of our non-resected patients with recurrent disease was relatively good at 34 months, but the OS after recurrence was not as good at 10 months. However, the median OS for patients with resection of recurrent disease was 83 months, and the median survival after recurrence was 30 months, which compares favourably with previous reports of systemic or local therapy.

Poor prognostic factors for DCC have been reported to include positive lymph node metastasis, positive portal vein invasion, positive perineural invasion, positive pancreatic invasion, and poor tumour differentiation [34–37]. Similarly, positive portal vein invasion and positive lymph node metastasis were independent poor prognostic factors in patients with primary DCC in our department. Kitano et al. stated that lymph node metastasis in cholangiocarcinoma is an independent poor prognostic factor in many reports and that patients with lymph node metastasis at the time of initial surgery should not be considered for resection of recurrent lesions [12]. In the cases of DCC resected in our department, R1 resection was not an independent poor prognostic factor, probably due to the inclusion of cases with positive marginal CIS. However, R1 cases had significantly more malignant findings on histopathology and significantly more local recurrences. R0 cases are considered more suitable for the resection of recurrent lesions than R1 cases.

Several reports have suggested that the time to recurrence is important in considering resection of recurrent cholangiocarcinoma lesions [9–13, 24]. In our study, the time to recurrence within one year is the strongest poor prognostic factor and is a crucial factor when considering the resection of recurrent lesions. In fact, a case of liver metastasis recurrence within one year was resected; however, the prognosis was not good as multiple liver metastases occurred within one year after resection. Several risk factors for early recurrence of distal cholangiocarcinoma have been identified. In a study of 245 resected cases, early recurrence within one year was associated with neutrophil-to-lymphocyte (NLR), peak total bilirubin, major vascular resection, lymphovascular invasion, and R1 resection. Patients were stratified according to the number of these risk factors, and the higher the number of risk factors, the significantly higher the early recurrence [25]. In a study of 486 cases, preoperative CA19-9, maximum tumour diameter, perineural invasion, and tumour differentiation were risk factors for early recurrence. The authors created a nomogram using these four factors and demonstrated that a higher score was associated with a significantly higher risk of early recurrence [38]. This means that the prognosis for patients with high risk factors for early recurrence is expected to be poor. Surgical resection would not be a treatment option, even if the recurrent lesion is resectable.

In addition, a few reports have mentioned how and when to introduce preoperative chemotherapy and the observation period before surgery when considering resection of recurrent lesions. Noji et al. advocated that at least three months of follow-up or chemotherapy induction should be performed for recurrent disease to select appropriate cases for metastasectomy [10]. In our study, no patient received preoperative adjuvant chemotherapy, and resection was performed early at the time of diagnosis of recurrence. Due to the small number of cases, future prospective studies are needed to clarify the necessity of preoperative adjuvant chemotherapy and the observation period required before resection.

As in previous reports, the resection of recurrent lesions in this study was performed safely without any complications requiring treatment [9, 12]. Takahashi et al. noted that the mortality rate was 1.4% in 74 resections of recurrent cholangiocarcinoma lesions, but the result was acceptable because of the relatively high number of difficult extended operations [9].

The indications for surgery according to the number and location of metastases have not been clarified. Yamada et al. reported 46 cases of pulmonary metastases from cholangiocarcinoma. Of the nine resected cases, four had two or more metastases, and three of the four patients died within two years. They stated that the indication for surgery in patients with multiple metastases should be carefully determined [13]. A multicentre retrospective study of 52 biliary tract cancer recurrent lesion resections reported that single distant metastases had a significantly better prognosis than local and combined recurrences, particularly for single liver metastatic recurrences, which may be a good indication for resection of recurrent lesions [27]. Similarly, this study also showed good outcomes in cases of resection of single recurrent liver metastases diagnosed more than one year after surgery. However, the number of cases in that study was small and it is not clear which cases were suitable for resection, as there were only seven cases and the oncological background of the patients, such as the recurrence site and the number of metastases, was heterogeneous compared to that of non-resected patients.

There are some limitations to this study. Firstly, it is a single-institutional retrospective study with a relatively small number of cases. It could be said that the results of this study would not be statistically reliable enough unless the total number of cases and the number of resected cases that recurred were much higher. Secondly, there is a possibility of selection bias in cases where recurrent lesions were resected. Further prospective multi-institutional studies are needed to clarify the effectiveness of resection in recurrent cholangiocarcinoma.

## Conclusion

This study investigated the efficacy of resection in recurrent lesions in DCC. Although the number of patients was limited, the results suggested the efficacy of resection in recurrent lesions in selected patients. Time to recurrence and single distant metastasis is considered an important factor; however, the small number of relevant cases in this study makes it difficult to conclude which patients are best suited for the resection of recurrent lesions. This issue requires clarification with a multicentre, prospective study, considering patients’ backgrounds, such as the recurrence site and the number of metastases.

## Data Availability

The datasets generated and/or analysed during the current study are available from the corresponding author upon reasonable request.

## Abbreviations

DCC: Distal cholangiocarcinoma
PD: Pancreaticoduodenectomy
PPPD: Pylorus-preserving pancreaticoduodenectomy
SSPPD: Subtotal stomach preserving pancreaticoduodenectomy
BDR: Bile duct resection
CIS: Carcinoma in situ
GEM: Gemcitabine
RFS: Recurrence-free survival
OS: Overall survival
BSC: Best supportive care
HR: Hazard ratio
CI: Confidence interval
